# Vagus nerve/recurrent laryngeal nerve ratio: proposal of a new parameter predicting left vocal cord palsy using intraoperative nerve monitoring during esophagectomy

**DOI:** 10.1007/s11748-025-02162-x

**Published:** 2025-05-28

**Authors:** Hiroyasu Ishikawa, Youichi Kumagai, Toru Ishiguro, Tetsuya Ito, Toshifumi Saito, Norimichi Chiyonobu, Noriyasu Chika, Takehiro Shiraishi, Takatoshi Matsuyama, Hideyuki Ishida

**Affiliations:** Department of Digestive Tract and General Surgery, Saitama Medical Center, Saitama Medical University, 1981 Kamoda, Kawagoe, Saitama 350-8550 Japan

**Keywords:** Esophageal cancer, Intraoperative nerve monitoring, Recurrent laryngeal nerve, Vocal cord palsy, V/R ratio

## Abstract

**Aim:**

Intraoperative nerve monitoring (IONM) during esophageal cancer surgery can help to identify and preserve the recurrent laryngeal nerve (RLN). To devise a useful parameter for prediction of left vocal cord palsy (VCP), we measured the electromyographic (EMG) amplitude of the left RLN and vagus nerve (VN) using intermittent IONM.

**Methods:**

We studied 35 consecutive patients who underwent esophagectomy with lymph node dissection around the left RLN. After lymph node dissection, the left RLN and left VN were stimulated, and the EMG amplitude was measured using IONM. The VN/RLN ratio (*V*/*R* ratio) was calculated, and the presence of left VCP, diagnosed by laryngoscopy on the first postoperative day, was compared among the patients.

**Results:**

Ten of the 35 patients (28.6%) had left VCP. In the VCP and non-VCP groups, the left VN amplitude was 190.0 (0–1111) µV and 520.0 (120–1200) µV (*P* = 0.006), and the VR ratio was 0.26 (0–0.75) and 0.71 (0.24–1.0) (*P* < 0.001), respectively. Receiver operating characteristic curve analysis using the left VN amplitude and V/R ratio showed an area under the curve (AUC) of 0.80 with a cutoff of 354 µV, and an AUC 0.90 with a cutoff of 0.50, respectively(*P* = 0.05). When left VN amplitudes of < 100 μV, < 354 μV, and a V/R ratio of ≤ 0.50 were defined as left VCP, the accuracy was 80.0%, 74.2%, and 88.6%, respectively.

**Conclusions:**

Using intermittent IONM, the V/R ratio with a cutoff value of 0.50 has the potential to be a more useful parameter for prediction of VCP after esophagectomy than EMG amplitude during VN stimulation.

## Introduction

Esophagectomy with extended mediastinal and cervical lymph node dissection has been shown to be beneficial for patients with resectable esophageal cancer, especially squamous cell carcinoma, which is the predominant type in Asia. Because of the high incidence of lymph node metastasis around the recurrent laryngeal nerve (RLN), lymphadenectomy around the RLN is one of the most important procedures during esophagectomy [1]. As a result, the incidence of RLN palsy (RLNP) is as high as 50% after radical esophagectomy with 3-field lymph node dissection [2, 3]. RLNP causes vocal cord palsy (VCP), and this results in not only hoarseness, but has also been reported as a risk factor for aspiration pneumonia [4, 5], even though the VCP is transient in most cases. As survival outcomes in patients who develop postoperative aspiration pneumonia are known to be poor [6–8], efforts should be made to prevent RLNP during esophagectomy. Recent technical advances have made it possible to identify the RLN during surgery using intraoperative nerve monitoring (IONM). This technique was first introduced for thyroid and parathyroid surgery [9–11] and has been reported to show high accuracy for detection of RLN during surgery, thereby reducing the incidence of RLNP significantly [12–16]. Similarly, IONM has been reported to be useful in the context of esophageal cancer surgery [17–25]. However, the relationship between IONM data and postoperative VCP has not yet been fully investigated, especially for the left RLN [18–20].

The present study examined the relationship between IONM data during esophagectomy and postoperative left VCP, with the aim of devising an appropriate parameter for prediction of postoperative VCP.

## Patients and methods

### Patients

We conducted a retrospective examination of 35 consecutive patients who underwent esophagectomy with lymphadenectomy around the left RLN (both thoracic and cervical lymphadenectomy) for esophageal or esophago-gastric malignant tumor at a single institution between August 2022 and September 2024. There were 29 men and 6 women with median age of 71 years (range 50–81 years); 26 patients underwent thoracoscopic surgery, 5 underwent robot-assisted surgery, and 4 underwent open thoracotomy. We performed bilateral cervical lymph node dissection including the paraesophageal (around the RLN) and supraclavicular area in 29 patients, whereas unilateral (left) cervical lymph node dissection was performed in 6 patients. The median operation time was 418 (237–559) min and the median blood loss was 275 (65–1225) ml. A gastric tube through the retrosternal route was used for reconstruction in 30 cases, and an ileo-colon through the ante-sternal route was used in 5 cases. According to the Union for International Cancer Control TNM staging system (8 th edition), the clinical stage of the tumor was Stage 0 in 1 patient, Stage 1 in 8, Stage 2 in 12, Stage 3 in 13, and Stage 4 in 1. The histological diagnosis was squamous cell carcinoma in 28 cases, adenocarcinoma in 6, and malignant melanoma in 1. The tumor was located in the upper thoracic esophagus in 7 cases, the middle thoracic esophagus in 16, the lower thoracic esophagus in 10, and the esophago-gastric junction in 2.

### Equipment setup and surgical procedure

Standard general anesthesia was used during surgery in all patients, who were intubated with Medtronic Nerve Integrity Monitor TriVantage® Electromyography (EMG) endotracheal tubes (Jacksonville, FL, USA) fitted with two exploratory electrodes above the cuff. Anesthesiologists used laryngoscopy to confirm that the exploratory electrodes adhered to the vocal cord. One-lung ventilation using a blocker inserted into the right bronchus was utilized. No muscle relaxant agent was used after initial induction of anesthesia. Before the IONM procedure, muscle relaxants were neutralized using antagonistic drugs in most cases. The NIM-response® system 3.0 (Medtronic Inc., Jacksonville, FL, USA) was used for IONM and a Prass® monopolar flush-tip probe (Medtronic Inc.) was used for nerve stimulation. This intermittent IONM (i-IONM) device makes it possible to identify the RLN as a wave displayed on the monitor upon direct stimulation of the RLN with the probe at 1.0 mA.

**Fig. 1:**
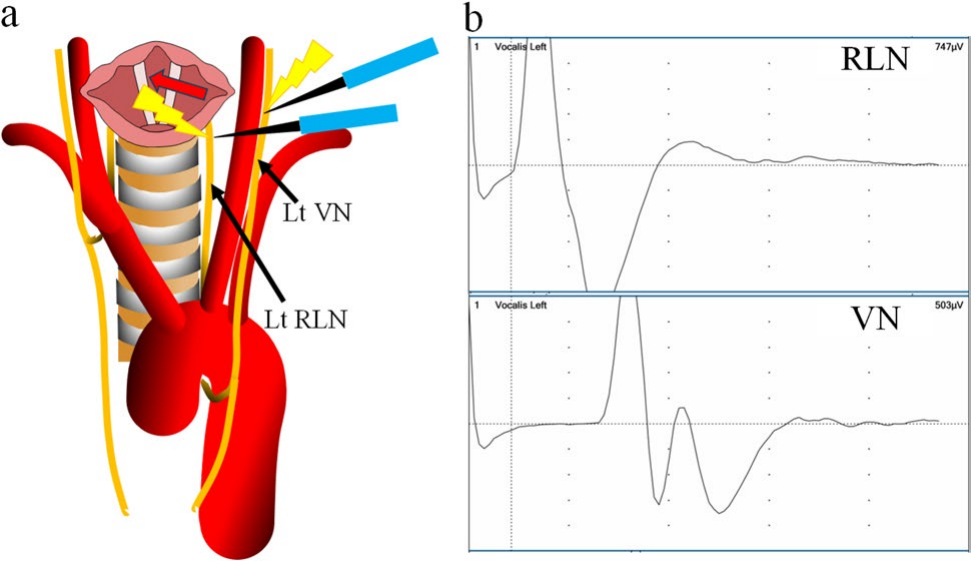
**a** Schema showing the stimulating points used in this study. We stimulated the left recurrent laryngeal nerve (RLN) and left vagus nerve (VN) during the cervical procedure. **b** Waveforms of the left VN and RLN. Electromyographic amplitudes for left RLN and VN stimulation were 747 µV and 503 µV, respectively. The calculated V/R ratio was 0.67

We performed the thoracic procedure with the patient prone or in left lateral recumbency prior to the cervical procedure. When the RLN was identified using i-IONM, lymph node dissection around the RLN was performed with reference to the i-IONM response. After the thoracic procedure, we changed the patient’s position to supine for the cervical procedure. In all cases, we dissected the left para-esophageal lymph nodes and exposed the cervical part of the left RLN and vagus nerve (VN). During cervical lymph node dissection, we identified the RLN and performed lymph node dissection around it by reference to the i-IONM responses of both the RLN and VN (Fig. 1a).

We stimulated the RLN and VN for several seconds and recorded the highest EMG amplitude for both after cervical lymph node dissection. The V/R ratio, i.e., the EMG amplitude upon VN stimulation divided by the EMG amplitude upon RLN stimulation, was then calculated (Fig. 1b). Loss of signal (LOS) was defined as < 100 µV when the VN was subjected to 1 mA of IONM stimulation, in accordance with the International Neural Monitoring Group guideline [9].

### Postoperative vocal code evaluation

All patients were extubated on the first postoperative day. The motility of the vocal cord was evaluated with a laryngoscope at the time of extubation by the pleural surgeons and anesthesiologists (more than three). VCP was diagnosed by mutual consensus. VCP was defined as the cessation or significant impairment of vocal cord movement compared to the preoperative endoscopic examination. The severity of VCP was assessed using the Clavien–Dindo (CD) classification system, which is widely used to grade postoperative complications [26]**.**

### Follow-up for patients

All patients routinely underwent upper gastrointestinal endoscopy and a vocal cord check at 1 year after the operation. During follow-up, VCP patients underwent laryngoscopy when hoarseness improved, and we determined the VCP recovery times (number of days from surgery until laryngoscopic confirmation of VCP recovery).

### Statistical analysis

Continuous variables were expressed as the median and range. The Mann–Whitney *U* test was used to assess differences between continuous variables, and Fisher’s exact test was used for categorical variables. To determine the cutoff values for prediction of postoperative RLNP, receiver operating characteristic (ROC) curve analysis was performed. Spearman’s rank correlation coefficients were estimated to evaluate relationships between two variables. Differences at *P* < 0.05 were considered statistically significant. StatFlex version 7.0 (Osaka, Japan) was used for all statistical analyses.

All procedures performed in the present study involving human participants were conducted in accordance with the 1964 Helsinki Declaration and its later amendments or comparable ethical standards.

Opt-out consent was obtained from all patients, and the study was performed under a protocol approved by our hospital ethics committee (2024-116).

## Results

During the study period, left VCP occurred in 10 patients (28.6%; CD-1 in 6, CD-2 in 2, and CD-3 in 2). The patient backgrounds according to the presence or absence of left VCP are shown in Table 1.

**Table 1:** Patient backgrounds according to the presence or absence of left vocal cord palsy

		Left VCP (*n* = 10)	Non-left VCP (*n* = 25)	*P* value
Age		69.5 (50–80)	71 (50–81)	0.956
Sex	Male	9	20	
	Female	1	5	0.65
Tumor location	Ut, Mt	10 (100%)	12 (48%)	
	Lt, EGJ	0 (0%)	13 (52%)	0.005
Clinical Stage	0, 1	3 (30%)	6 (24%)	
	2, 3, 4	7 (70%)	19 (76%)	0.69
Operation	MIS	8 (80%)	23 (92%)	
	Thoracotomy	2 (20%)	2 (8%)	0.56
Operation time (min)		417 (323–559)	418 (237–545)	0.63
Blood loss (ml)		194 (65–1225)	296 (83–625)	0.15
Post operation pneumonia		1 (10%)	1 (4%)	0.49
Anastomotic leakage		1 (10%)	1 (4%)	0.49
Preoperative lymph node metastasis along the RLN		1 (10%)	3(12%)	1.0

*Ut* upper thoracic esophagus, *Mt* middle thoracic esophagus, *Lt* lower thoracic esophagus, *EGJ* esophagogastric junction, *MIS* minimally invasive surgery

Only tumor location was significantly correlated with left VCP (*P* = 0.005); no other parameters, including postoperative pneumonia and preoperative lymph node metastasis along the RLN, showed a statistically significant difference between the VCP and non-VCP groups.

**Fig. 2:**
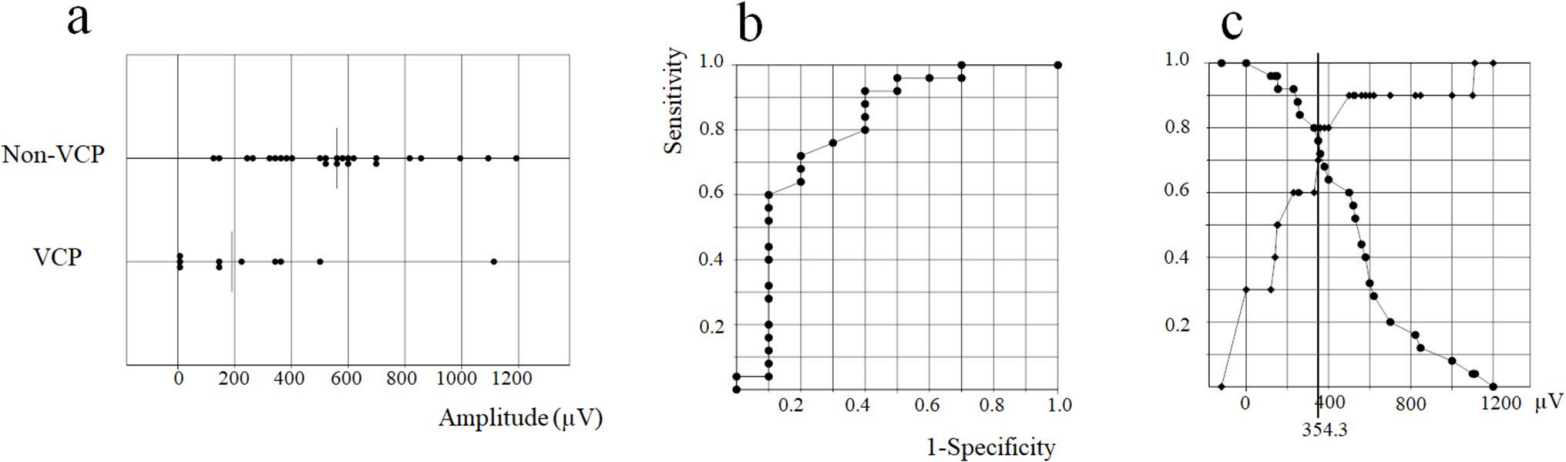
**a** Electromyographic amplitude for VN stimulation in the left vocal cord palsy (VCP) group and the non-left VCP group (*P* = 0.006). **b** Receiver operating characteristic curve analysis for the left VCP plotted using the EMG amplitude for vagus nerve stimulation. Area under the curve was 0.80. **c** Sensitivity and specificity of the curve for diagnosis of VCP using the EMG amplitude for VN stimulation (●: sensitivity, ◆: specificity). The cutoff value that revealed the highest sensitivity and specificity was 354 µV

The median EMG amplitude for the left VN in the non-left VCP group (520.0 µV; 120–1200 µV) was significantly higher than that in the left VCP group (190.0 µV; 0–1111 µV) (*P* = 0.006) (Fig. 2a).

ROC curve analysis of the left VCP plotted using the EMG amplitude for the left VN showed that the area under the curve (AUC) was 0.80 (Fig. 2b). The cutoff value with the highest sensitivity and specificity from the ROC analysis was 354 µV (Fig. 2c).

**Fig. 3:**
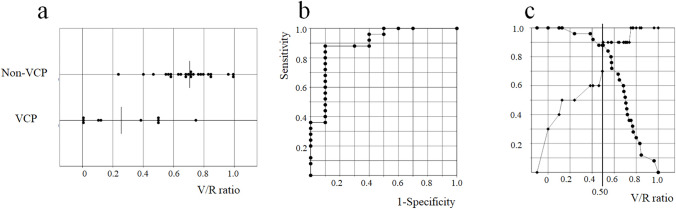
**a** *V*/*R* ratio for the left vocal cord palsy (VCP) group and the non-left VCP group (*P* < 0.001). **b** Receiver operating characteristic curve analysis for the left VCP plotted using the *V*/*R* ratio. Area under the curve was 0.90. **c** Sensitivity and specificity curve for diagnosis of VCP using the *V*/*R* ratio (●: sensitivity, ◆: specificity). The cutoff value of the *V*/*R* ratio that revealed the highest sensitivity and specificity was 0.50

The median V/R ratio in the non-left VCP group (0.71; 0.24–1.00) was significantly higher than that in the left VCP group (0.26; 0–0.75) (*P* < 0.001) (Fig. 3a).

ROC curve analysis of the left VCP plotted using the V/R ratio showed that the AUC was 0.90 (Fig. 3b), and that the cutoff value with the highest sensitivity and specificity was 0.50 (Fig. 3c).

The difference between the AUC for the ROC curve plotted using the EMG amplitude of the left VN and that for the ROC curve plotted using the V/R ratio tended to be significant (*P* = 0.05).

When the patients were divided into two groups with EMG amplitudes of < 100 µV (LOS) and ≥ 100 µV for VN stimulation, the sensitivity, specificity, positive predictive value, negative predictive value, and accuracy for prediction of left VCP were 30.0%, 100%, 100%, 78.1%, and 80.0%, respectively, and when the patients were divided into two groups with EMG amplitudes of < 354 µV and ≥ 354 µV for VN stimulation, the corresponding values were 53.8%, 86.4%, 70.0%, 76.0%, and 74.2%, respectively. When the patients were divided into two groups with V/R ratios of ≤ 0.50 and > 0.50, the sensitivity, specificity, positive predictive value, negative predictive value, and accuracy for prediction of left VCP were 90.0%, 88.0%, 75.0%, 95.7%, and 88.6%, respectively (Table 2).

When VCP patients were divided into the CD-1 group and the CD-2,3 group, the V/R ratios were 0.43 (0–0.75) and 0.05 (0–0.5), respectively. There was no statistically significant difference between the groups (*P* = 0.28). The amplitude of left VN stimulation in VCP patients was 296 µV (0–1111) in the CD-1 group and 75 µV (0–350) in the CD-2,3 group, with no statistically significant difference observed (*P* = 0.25).

**Table 2:** Presence or absence of left vocal cord palsy (VCP) in relation to electromyographic amplitude cutoff values of 100 µV and 354 µV for vagus nerve (VN) stimulation and a *V*/*R* ratio cutoff value of 0.50

	Left VCP	Non-left VCP
amplitude of VN stimulation < 100 µV	3	0
amplitude of VN stimulation ≥ 100 µV	7	25
amplitude of VN stimulation < 354 µV	7	6
amplitude of VN stimulation ≥ 354 µV	3	19
*V*/*R* ratio ≤ 0.50	9	3
*V*/*R* ratio > 0.50	1	22

**Fig. 4:**
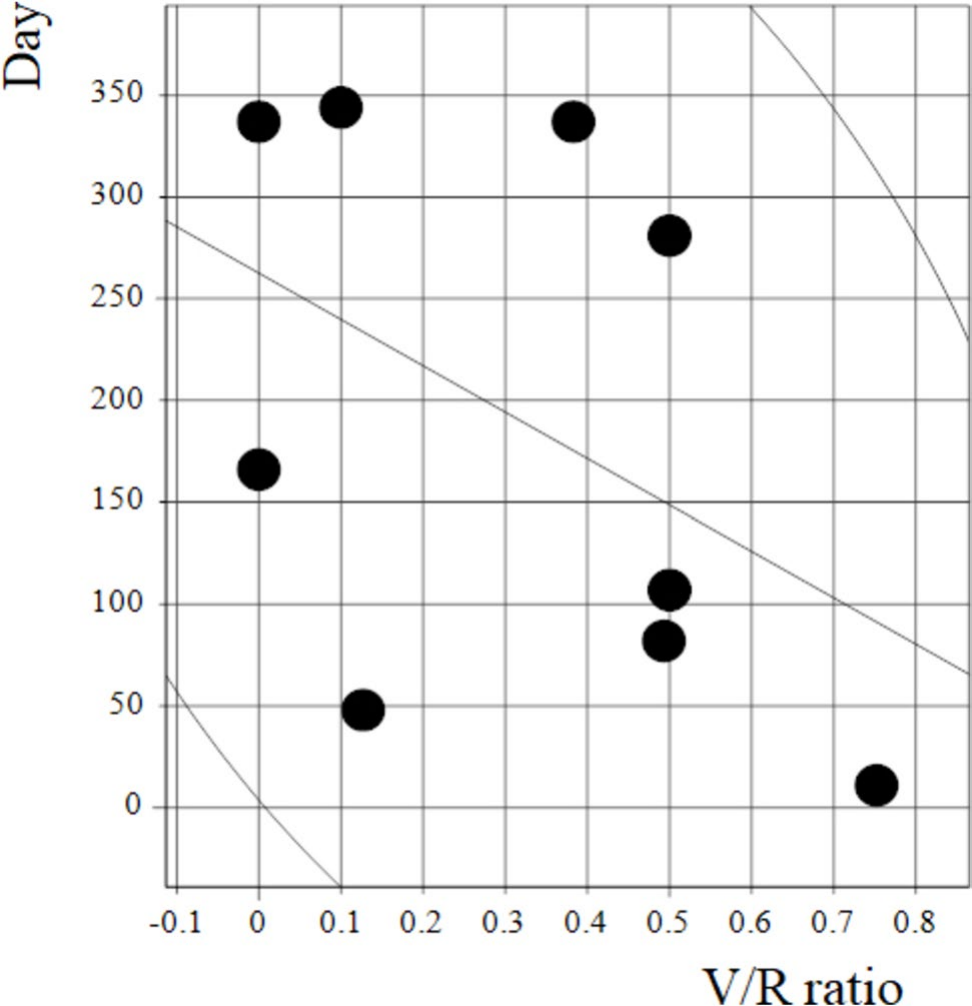
Correlation between the *V*/*R* ratio and vocal cord palsy recovery time (rS = −0.53, *P* = 0.18)

Nine of the 10 patients with left VCP recovered within a year. One patient was lost to follow-up. There was a relatively strong correlation between V/R ratio and VCP recovery time, but the *P* value did not reach significance (rS = −0.54, *P* = 0.14) (Fig. 4).

## Discussion

In the present study, we investigated a new parameter for prediction of left VCP using i-IONM—the V/R ratio—and found that it had high diagnostic accuracy, being superior to the EMG amplitude of VN stimulation (i.e., conventional LOS of the i-IONM, which uses an amplitude of < 100 µV as the cutoff, or a cutoff value of 354 µV calculated using ROC curve analysis).

In esophageal cancer surgery, there are several problems with the conventional method for prediction of RLNP based on EMG amplitude for VN stimulation. First, the amplitude varies between patients; if the electrode is not properly placed on the vocal cords, the amplitude will decrease [9]. Changes in patient during esophagectomy (from prone or left lateral recumbency to supine) may also dislocate the electrode and thus influence the amplitude. Therefore, the amplitude obtained from RLN stimulation during thoracic procedures cannot be compared with the amplitude obtained during cervical procedures. Furthermore, muscle relaxants are known to influence amplitude [24]. Second, in esophagectomy, the thoracic procedure is performed first. Even if the vocal cords respond to VN stimulation using i-IONM during the cervical procedure, RLN might have already been injured during the thoracic procedure. Third, the amplitude during VN stimulation changes due to surgical maneuvers such as traction. Therefore, when examining the relationship between amplitude during VN stimulation and postoperative VCP, it is necessary to compare it with the amplitude measured after the completion of lymph node dissection around the RLN. Furthermore, the timing of VCP assessment is also important. We evaluate vocal cord movement immediately after extubation on postoperative day 1 and compare it with the V/R ratio after lymph node dissection around the RLN. Checking for VCP at the time of extubation is important, as it allows for early intervention in cases of severe VCP, such as bilateral RLNP that causes airway obstruction. Some previous studies have reported evaluating vocal cord function 1 week after surgery [11, 19, 21, 23].  If RLNP can be accurately predicted during surgery, it becomes possible to prepare for these serious complications before extubation.

In the present study, 70% of the left VCP cases had an EMG amplitude of > 100 µV during VN stimulation, and if an amplitude of 100 µV was used as the cutoff, these cases would have been judged as having no RLNP. ROC curve analysis showed that when a VN stimulation cutoff value of 354 µV was used, the overall accuracy for prediction of left VCP (74.2%) was not satisfactory, nor was a cutoff value of 100 µV (80.0%). Clearly, therefore, another parameter that reflects the baseline EMG amplitude of VN before injury to the RLN is needed. The EMG amplitude at the most distal side evoked by RLN stimulation during the cervical procedure is considered to approximate the baseline EMG amplitude for unimpaired VN stimulation. Thus, the V/R ratio may reflect the degree of injury of the RLN more precisely than the EMG amplitude upon VN stimulation.

IONM employs another probe that continuously stimulates the VN (c-IONM) [9, 14, 15, 24, 25]. c-IONM can visualize the gradual decrease in amplitude due to traction or compression of the RLN under continuous stimulation of the VN, and is reported to help avoid intraoperative RLN injury [14, 15, 24, 25]. In terms of esophagectomy for esophageal malignancies, which requires full-length exposure of the RLN and lymph node dissection, both i-IONM and c-IONM have their own advantages. We use i-IONM, rather than c-IONM, during both thoracic and cervical procedures, because precise identification of the RLN using i-IONM is beneficial. Despite the use of i-IONM in this study, the incidence of left VCP was relatively high at 28.6%. RLNP can result from sudden physical impacts, such as thermal injury, excessive grasping, or inadvertent traction. While i-IONM is useful for identifying RLN, it does not enable real-time monitoring of progressive nerve injury. Therefore, when using i-IONM, meticulous dissection around the nerve after its identification is essential to minimize the risk of VCP. Incorporating c-IONM in future studies may offer intraoperative warnings during surgical maneuvers that could lead to RLN injury, potentially reducing the incidence of VCP.

The cutoff value of the *V*/*R* ratio for prediction of left VCP was set at 0.50. The International Neural Monitoring Group guidelines [9] consider that a 50% decrease or more from the baseline VN stimulation amplitude when using c-IONM indicates serious RLN injury. A V/R ratio cutoff value of 0.50, calculated using i-IONM, can be considered equivalent to a cutoff value of 50% from baseline VN stimulation using c-IONM, and correlates well with postoperative VCP. During the thoracoscopic procedure, Fujimoto et al. [21] reported that all cases with an attenuation rate exceeding 60% of EMG amplitude determined using i-IONM between the most proximal and most distal sides of the left RLN showed RLNP postoperatively. As described above, when using i-IONM, the amplitude attenuation rate at these two distant points is important for RLNP assessment. Furthermore, reduction of EMG amplitude by approximately half is a sign of severe RLN injury.

In the present study, there was one case of VCP with a *V*/*R* ratio of 0.75. In this case, hoarseness improved within a week, and laryngoscopy on postoperative day 11 confirmed that VCP had improved.

The *V*/*R* ratio and VCP recovery time showed a relatively strong correlation, although it was not significant. In addition, the severity of VCP, as assessed using the Clavien–Dindo classification, tended to be greater with lower *V*/*R* ratios; however, this difference was also not statistically significant. These findings suggest that RLNP with a lower *V*/*R* ratio tends to be more severe and may require a longer recovery period. Further studies with larger case numbers are needed to validate these findings. There were also three cases in which the *V*/*R* ratios were < 0.50 without VCP. When evaluating the response to VN stimulation during the cervical procedure, we stimulate multiple points of the VN and search for the highest point of the EMG amplitude. These three cases showing a low *V*/*R* ratio without RLNP might have been attributable to the probe not making proper contact with the fiber bundle of the RLN in the main trunk of the VN.

The present study has several limitations. First, it was a single-center study involving a small number of cases. The cutoff value of the V/R ratio will need to be verified by examining a large number of cases at multiple institutions in a prospective study setting. Second, this study examined only left VCP. In a future study, it would be interesting to investigate whether right VCP would yield similar results.

In conclusion, when using i-IONM, the *V*/*R* ratio and a cutoff value of 0.50 has the potential to be a more useful parameter for prediction of VCP after esophagectomy than the conventional parameter of EMG amplitude during VN stimulation.

## Data Availability

The date that support the findings of this study are available from the corresponding author, YK, upon reasonable request.
